# Preparation of Permeable Porous Alumina Ceramics by Gel Casting Combined with Particle Stacking and Sintering Method

**DOI:** 10.3390/ma18153463

**Published:** 2025-07-24

**Authors:** Zhe Cheng, Yuanqing Chen, Zhenping Wu, Yang Liu

**Affiliations:** 1 School of Material Engineering, Shaanxi Polytechnic University, Xianyang 712000, China; 13809101910@163.com; 2 School of Materials Science and Engineering, Xi’an University of Technology, Xi’an 710048, China; chenyq@xaut.edu.cn (Y.C.); 13022869971@163.com (Z.W.)

**Keywords:** gas permeability, ceramics, porous materials, gel casting

## Abstract

Porous ceramics have been widely used in various fields. In this paper, porous ceramics with through-hole structures were prepared using a novel and eco-friendly gel casting method with carrageenan as the gelling agent. Especially, the idea of large size particle stacking is introduced into the gel casting process. By introducing large size alumina aggregates as raw materials, and small size micropowders as filling materials, micropores were directly formed after the green body was sintered. To tune the pore size, pore structure, gas permeability, the strength of the final porous ceramics, the components of the raw materials including the alumina aggregates, the filling materials, and sintering additives in the slurry were precisely designed. Porous Al_2_O_3_-based ceramics with high gas permeability, high flexural strength, and moderate porosity were finally obtained.

## 1. Introduction

Nowadays, porous ceramics have been widely used in various fields, including water treatment, catalyst support, sound absorption, and so on. The application of porous ceramics is closely related to the pore size and pore structure. For example, the porous ceramics with open pore sizes ranging from 100 to 1000 μm can be used in sound absorption, physical filtration, and thermal insulation [1,2,3], while those with open pore sizes of 1~100 μm are widely used in bacteria culture, catalyst support, vacuum chunk, and so on [4,5,6].

Many methods have been developed to prepare porous ceramics, including freeze casting [7], sacrificial templating [8,9], direct foaming [10], and, in the latest years, additive manufacturing (3D printing) [11]. Freeze casting can easily obtain ceramics with through-hole structures. Sacrificial templating and, more particularly, emulsion templating as well as foaming have proved to be of keen interest for the elaboration of materials with tunable porosity. Very recently, techniques like foaming or sacrificial templating have been combined with 3D printing to produce porous ceramics [12]. This new technique can directly create porous structures like lattices. Especially, complex structures can be pre-designed, which are then completed (fabricated) via 3D printing [13]. However, most of these methods cannot realize ceramics with interconnected open pores with a pore size of less than several tens of micrometers.

It is known that the gel casting method can also be used to prepare porous ceramics. In the gel casting process, acrylamide or methyl acrylamide were often used as starting agents [14,15]. To avoid toxicity, some green and non-toxic natural gel systems (such as natural gum, ISOBAM, and so on) have also been used in the gel casting route [16,17]. Nowadays, the gel casting technique has been widely investigated and used to prepare different kinds of ceramics in different applications [18,19,20]. Recently, the gel casting route was developed to prepare porous ceramics by combining other processes, e.g., the foaming method. This method can produce porous ceramics with a porosity as high as 80~90% [21]. However, their pore size was always higher than 100 μm. Moreover, in the gel casting route, toxic acrylamide as a raw material is often used, limiting its large-scale production. On the other hand, by introducing a sacrificial template, gel casting can be used to produce pores with sizes of several or several tens of micrometers [22]. However, this method is not environmentally friendly.

In addition to gel casting, the direct stacking and sintering method for the preparation of porous ceramics was recently reported [23,24]. Interestingly, this method does not need pore-forming agents or sacrificial templates. In this method, both the large-size and small-size powders were used as raw materials, which were then dry pressed and sintered. Through-pores with a size of several micrometers were directly formed during the sintering process.

Inspired by this, we realize that if large-size particles are introduced as raw materials in gel casting, the porous ceramics with through-pores would be obtained without the introduction of pore-forming agents or sacrificial templates. Moreover, the pore size and porosity of the porous ceramics would be easily tuned.

Intrigued by this idea, in this paper, we developed a new and low-cost gel casting method to prepare porous ceramics with through-holes, which is of potential application in the fabrication of large-size ceramic sheets used for vacuum chunk. To meet the requirement of the ceramic chunk, we fully take into account the design of the component, the selection of the raw materials, and the microstructure of the products. Especially, to avoid the use of the toxic acrylamide, a new gel casting process was developed using carrageenan as a crosslinking agent. The finally obtained porous ceramic shows a high strength over 160 MPa, a high porosity over 30%, and a high permeability of ~36 L/(min·cm^2^).

## 2. Materials and Methods

**Figure 1:**
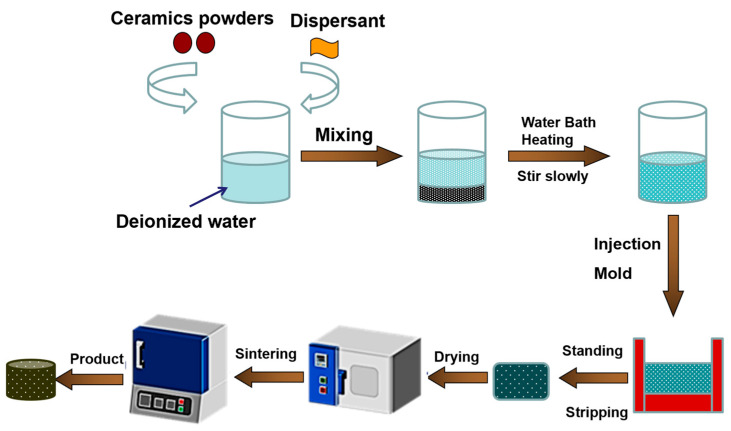
Experimental process for preparation of the porous ceramics.

The experimental process for the preparation of porous ceramics is shown in Figure 1. In a typical experiment, 1.5 g ammonium polyacrylate was added into 64 g deionized water. Then, 0.5 g KCl and 0.5 g carrageenan were added into the solution. After that, the pH of the solution was adjusted to 8 with ammonia. Subsequently, 150 g ceramic powders were slowly added to deionized water and stirred at 80 °C for 10 min. The prepared ceramic slurry was then poured into a mold constructed by glass sheets. After the slurry was cooled to room temperature, the slurry was self-solidified. The obtained green bodies with a thickness of about 5 mm were then dried at 60 °C for 12 h and then sintered at 1500 °C for 4 h. The ceramic powders were composed of 10 wt.% kaolin powders (4000 mesh), 5 wt.% sintering aids (Y_2_O_3_/MgO = 8/2, *w*/*w*), and 85 wt.% alumina powders. Particularly, the alumina powders consisted of large-size Al_2_O_3_ aggregate particles (200~1000 mesh) and small-size active α-Al_2_O_3_ micropowders (1~3 μm). All chemical reagents are of analytical purity.

The microstructure of the samples was observed by a scanning electron microscope (JSM-6700, JEOL, Tokyo, Japan). Before the microstructure observation, a thin layer of Au metal was sputtered on the sample surface so as to enhance the sample conductivity. During observation, the working distance was 20 mm and electron beam energy was kept at 2 kV. The porosity and bulk density were measured by Archimedes’ method. The flexural strength was tested by a three-point bending method under a universal testing machine (HT-2402, Hung Ta Instrument Co., Ltd., Taiwan, China). The length of the test sample was controlled at 50 mm, with a cross-section area of 3 mm × 4 mm. During the test, the span of the sample was 30 mm, and the loading rate was controlled at 0.4 mm/min. The pore size and the size distributions were measured using mercury intrusion porosimetry (Auto Pore V9600, Micrometrics, Norcross, GA, USA). The gas permeability of the porous ceramics was tested by self-made permeability testing equipment, and N_2_ was selected as the gas source. Before testing, the samples were polished into pieces with a diameter of 50 mm and thickness of 6 mm.

## 3. Results and Discussion

### 3.1. Mechanism of Forming Through-Holes by Gel Casting Combined with Particle Stacking and Sintering Method

**Figure 2:**
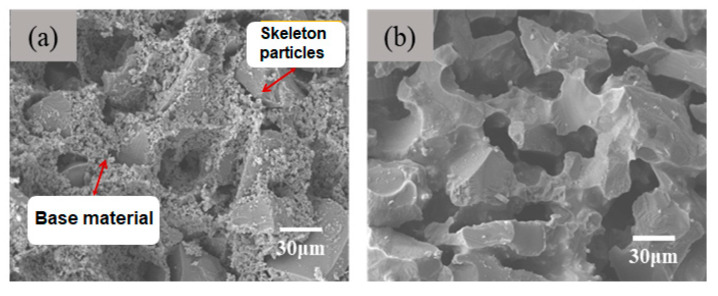
Comparison of morphology before and after sintering: (**a**) before, (**b**) after.

In this paper, large-size white corundum is used as ceramic aggregate, and the α-Al_2_O_3_ micropowder is the matrix (binders). The method of combining gel casting with particle stacking and sintering is used to successfully build porous ceramics. The results of a typical sample are shown in Figure 2.

**Figure 3:**
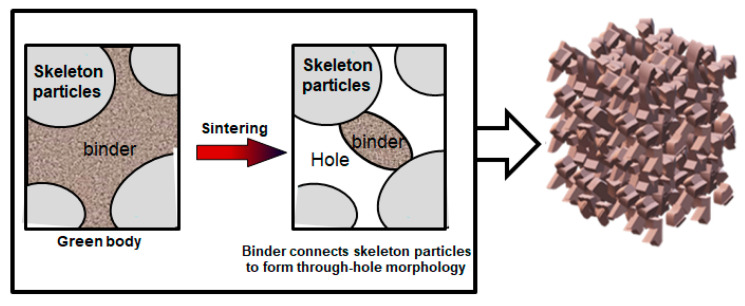
Schematic diagram of ceramics with interconnected open-pore structure.

In the green body, the ceramic matrix is covered on the aggregate surface or filled in the pores formed by particle stacking. During the sintering process, the matrix is more prone to shrinkage due to the volume effect between the matrix and the aggregate. After shrinkage, the matrix forms a sintering neck, connecting the aggregate particles. And the pore structure is directly formed by aggregate accumulation. The gel casting method enables the particles to loosely accumulate, and the matrix and aggregate distribute more uniformly. Therefore, the final porous ceramics have a large number of interconnected pore structures. As indicated by Figure 3, the aggregates and binders (matrix) are loosely stacked in the green body. After sintering at a certain temperature, large particle aggregates of the ceramics are connected by binders. Since only a few points are connected, it presents a three-dimensional through-hole morphology.

### 3.2. Influence of Aggregate–Matrix Ratio on Morphological Structure and Properties of Through-Porous Ceramic Materials

It is known that the pore size and porosity of porous ceramics are influenced by many factors when the gel casting method is used. In our work, the large-size Al_2_O_3_ aggregates and α-Al_2_O_3_ micropowders were introduced in the slurry. Obviously, in addition to the solid content of the slurry, the size of the Al_2_O_3_ aggregate and its content show great influence on the pore structure and pore size. Therefore, the component design is of great importance to tune the properties of the porous ceramics. To investigate the effect of the weight ratio of the Al_2_O_3_ aggregate to α-Al_2_O_3_ micropowders on the pore structure of the porous ceramics, five samples with different Rm (Rm = 10 wt.%, 20 wt.%, 30 wt.%, 40 wt.%, and 50 wt.%) were prepared. Here, Rm = Wm/(Wa + Wm), where Wm and Wa refer to the weight of Al_2_O_3_ aggregates and α-Al_2_O_3_ micropowders, respectively. The raw materials designed for the five samples are listed in Table 1. The size of the Al_2_O_3_ aggregates in the slurry was kept the same (320 mesh) for all samples, and the solid content of the slurry was fixed at about 70 wt.% (~40 vol.%).

**Figure 4:**
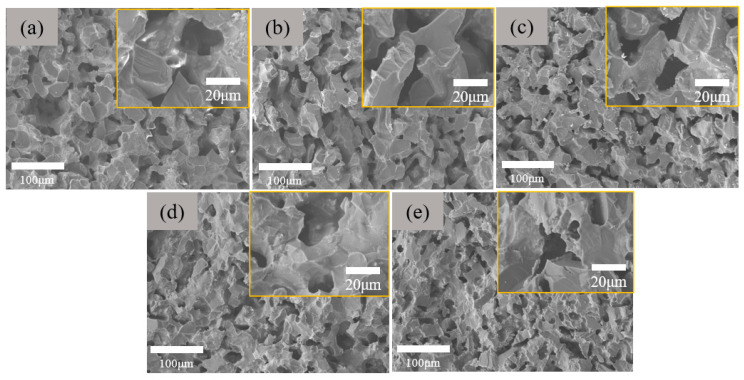
Morphologies of porous ceramics prepared with different Rm: (**a**) 10%, (**b**) 20%, (**c**) 30%, (**d**) 40%, and (**e**) 50%.

**Figure 5:**
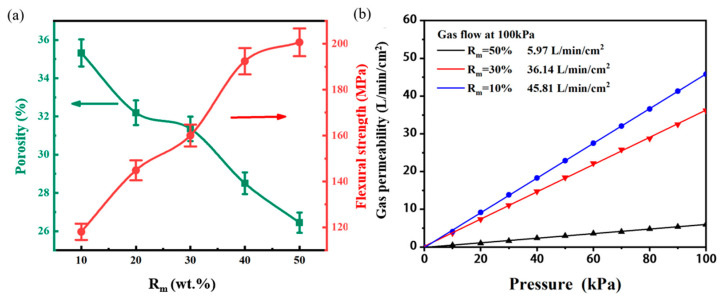
(**a**) Relationship between different Rm ratios and flexural strength and porosity. (**b**) Relationship between different matrix ratios and permeability.

The morphologies of the inner structure of the as-prepared porous ceramics are shown in Figure 4. Basically, the morphologies of all the samples are almost the same. A careful investigation discloses that with the increase in the Rm ratio from 10 wt.% to 50 wt.%, the pore size slightly decreases. The open porosity and flexural strength of the samples are shown in Figure 5a. As predicted, with the increase in the Rm, the open porosity decreases, while the flexural strength increases almost linearly. These results can be explained as follows: In the green bodies, the pores are directly formed due to the stacking of the large-size Al_2_O_3_ aggregates, while the α-Al_2_O_3_ micropowders, together with kaoling powders and sintering aids, act as the pore fillings in the green body; therefore, with the increase in the content of α-Al_2_O_3_ micropowders, the pore size and porosity decreases. Moreover, during the sintering stage, the Al_2_O_3_ aggregates are almost inert, while α-Al_2_O_3_ micropowders, kaoling powders, and sintering aids tend to form the liquid phase [25,26], facilitating the crystallization of the Al_2_O_3_ phase, and thus improving the flexural strength. The gas permeability of the samples was further tested. As shown in Figure 5b, the gas permeability decreases gradually with the increase in Rm. This is in agreement with the result of open porosity. It is found that when the Rm = 30%, not only is the porosity of the sample higher than 30%, but its flexural strength is also nearly 160 MPa, and the gas permeability reaches ~36 L/min/cm^2^ at 100 KPa. All of these data indicate its potential application for vacuum chuck.

### 3.3. Influence of Aggregate Size on the Morphological Structure and Properties of Through-Porous Ceramic Materials

According to the above research results, the proportion of aggregate and matrix in powder is the key parameter to successfully build a porous ceramic with good performance and excellent pore structure when using the particle stacking method to prepare porous ceramic materials. However, how does the ratio of aggregate to matrix affect the bending strength? Because Al_2_O_3_ skeleton particles are inert during sintering, the formation of the pores will be related to the reaction of the filler material, including the active α-Al_2_O_3_ powder, kaolin powder, and sintering additives. It is well known that rare earth oxides Y_2_O_3_ and La_2_O_3_ are good surface-active substances. The addition of rare earth materials promotes the solid-state reaction between Al_2_O_3_ powder and the low melting point liquid phase. Therefore, the two substances play an important role in the formation of pore structure during sintering: one is inert Al_2_O_3_ particles (skeleton particles), and the other is a quasi-liquid phase.

**Figure 6:**
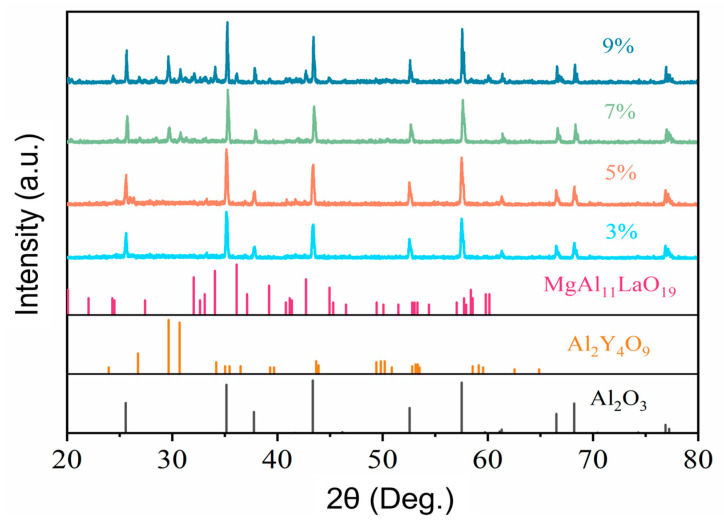
XRD patterns of sintered samples with different additives (3, 5, 7, and 9 wt.%).

To verify this, the samples with different amounts of aids are sintered at 1500 °C. The XRD diffraction patterns of samples with different amounts of sintering aids after sintering are shown in Figure 6. It can be seen that the XRD patterns of the samples with aids of 1~5 wt.% are basically consistent, and only the diffraction peaks of Al_2_O_3_ can be observed. And the intensity of the diffraction peaks increases with the content of the sintering aids. This indicates that under the same sintering temperature, the crystallinity of the sample increases with the content of the aids. When the additional amount of the sintering aids reaches 7 wt.%, small peaks corresponding to other phases appear at 2θ values of 29.65° and 30.70°. These second phases mainly correspond to Al_2_Y_4_O_9_ and MgAl_11_LaO_19_. These second phases will help the Al_2_O_3_ to form a quasi-liquid phase at the high sintering temperature. Considering the resolution limit of XRD, these phases may also exist in the samples with an additional sintering aid amount of less than 7 wt.%. It should be noted that the strength of the samples is closely related to the second phases (quasi-liquid phase), whereas the second phases or the quasi-liquid phases may not fully connect the aggregate, because it is related to the size and content of the aggregates.

**Figure 7:**
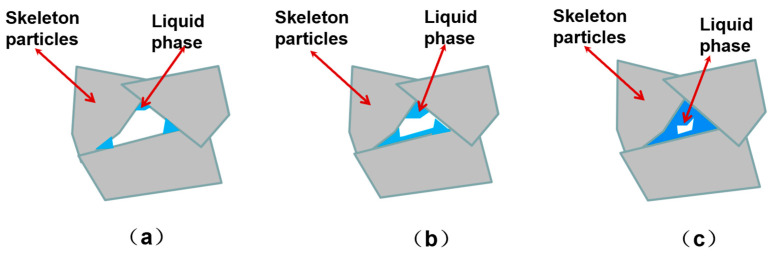
Schematic diagram of liquid phase formation in ceramics during sintering process: (**a**) low content of active α-Al_2_O_3_ powders; (**b**) medium content of active α-Al_2_O_3_; and (**c**) high content of α-Al_2_O_3_ powders.

Therefore, the effect of the ratio of the aggregate to matrix in the powder on the strength of porous ceramics can be explained as follows: when the content of active α-Al_2_O_3_ powders is low, the quasi-liquid phase may not be completely formed. The liquid phase preferentially fills the sharp corners of adjacent skeleton particles, thus reducing the surface energy of the system, as shown in Figure 7. However, because of the low content of liquid, the connection of the aggregates is not of high strength, as indicated by Figure 7a. With the increase in matrix content, the material is consumed and transformed into a large amount of the liquid phase, as shown in Figure 7b. Further increase in the amount of liquid phase will make the liquid phase tightly wrap and connect the skeleton Al_2_O_3_ powder, increasing its strength. However, the diameter of the hole caused by the adjacent skeleton Al_2_O_3_ particles will be decreased, as shown in Figure 7c. Therefore, with the increase in Rm, the strength of ceramics is increased, but the permeability of the porous ceramic will decrease if too much liquid phase is introduced.

**Figure 8:**
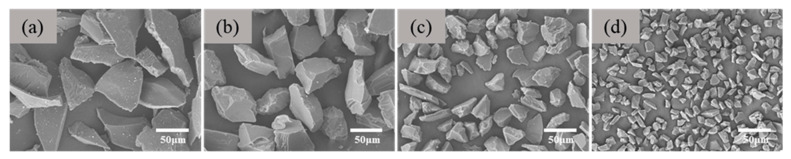
SEM images of white corundum (aggregate) with different mesh numbers: (**a**) 200 mesh, (**b**) 320 mesh, (**c**) 600 mesh, and (**d**) 1000 mesh.

To further tune the pore structure of the porous ceramics, we prepared porous ceramics using Al_2_O_3_ aggregates of 200, 320, 600, and 1000 mesh (about 81.3, 51.8, 28.5, and 14.8 μm), respectively. Figure 8 shows the scanning electron images of white alumina aggregates with a particle size of 200, 320, 600, and 1000 mesh. Table 2 shows the statistical results of the particle size. It can be found from the chart that the particle size distribution of the white corundum powder used is relatively uniform, and the particles are irregular. The larger the mesh number, the smaller the average size.

**Figure 9:**
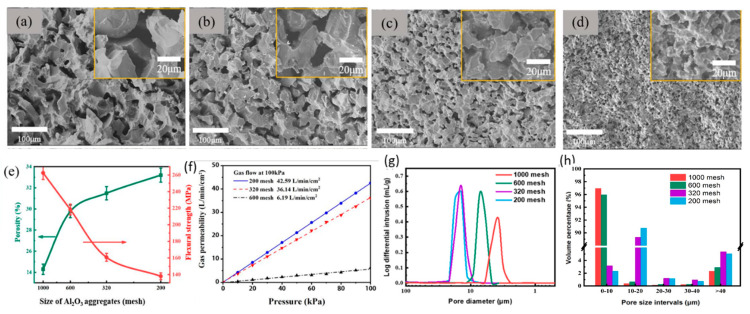
Morphologies of porous ceramics prepared using Al_2_O_3_ aggregates with different sizes: (**a**) 200 mesh, (**b**) 320 mesh, (**c**) 600 mesh, and (**d**) 1000 mesh. Properties of porous ceramics: (**e**) porosity and flexural strength of the samples; (**f**) gas permeability of samples; (**g**) pore size distribution; and (**h**) volume percentage of pores.

Using the Al_2_O_3_ aggregates with different sizes, porous ceramics were prepared. The morphologies of the inner structure are shown in Figure 9a–d, and their properties are shown in Figure 9e–h. With the size of Al_2_O_3_ aggregates decreasing from 1000 mesh to 200 mesh, the pore size and porosity gradually decreases. Conversely, the flexural strength increases from ~140 MPa to ~260 MPa. The results shown in Figure 9f indicate the permeability shows a negligible drop when the Al_2_O_3_ aggregate size decreases from 200 mesh to 320 mesh, but it shows a dramatic drop from ~36 to ~6 L/min/cm^2^ when the Al_2_O_3_ aggregate size decreases from 320 mesh to 600 mesh, which is related to the pore structure.

The pore structure of the samples was examined by an automatic mercury porosimeter. Their results are shown in Figure 9g. The average pore size is about 15 μm when 200 or 320 mesh aggregates are used. Decreasing the size of the aggregates to 600 and 1000 mesh leads to the average pore size of about 7 μm and 4 μm. The volume percentage of pores with different sizes is shown in Figure 9h. For samples prepared using 600 and 1000 mesh aggregates, the pores with a size of 0~10 μm take up the volume percentage of more than 95%, while in samples prepared using 200- and 320-mesh aggregates, 90% of the volume percentage is taken up by pores with a size of 10~20 μm. These results explain the reason why the permeability is almost the same when the size of Al_2_O_3_ aggregates decreases from 200 mesh to 320 mesh but shows a dramatic drop when the size of Al_2_O_3_ aggregates decreases from 320 mesh to 600 mesh.

## 4. Conclusions

A novel and environmentally friendly gel casting method using carrageenan as a gelling agent was developed to prepare porous ceramics. Especially, the idea of particle stacking is used to construct a through-hole structure. The component raw materials including large-size Al_2_O_3_ aggregates and active α-Al_2_O_3_ micropowders were concisely designed. The micropores were directly formed by stacking the large size Al_2_O_3_ aggregates during the sintering process. Assisted by the gel casting method, the loosely accumulated aggregate helps the formation of the through-hole structure. The filling materials of Al_2_O_3_ micropowders and sintering aids connected the Al_2_O_3_ aggregates and enhanced the flexural strength of the porous ceramics. By tuning the weight ratio of the Al_2_O_3_ aggregates and α-Al_2_O_3_ micropowders, a porosity with both high gas permeability and high flexural strength can be obtained. We found that when the Rm reaches 30% in the slurry, porous Al_2_O_3_-based ceramics with a porosity over 30%, gas permeability over 36 L/min/cm^2^, and high flexural strength of ~160 MPa were obtained.

## Figures and Tables

**Table 1:** The content of the raw materials used for preparation of different samples.

Samples	Rm	Al_2_O_3_ (320 mesh)	α-Al_2_O_3_ (1–3 μm)	Kaoling Powders (4000 mesh)	Sintering Aids		
					Y_2_O_3_	La_2_O_3_	Mg_2_(OH)_2_CO_3_
A	10%	114.75 g	12.75 g	15.00 g	3.75 g	2.25 g	1.5 g
B	20%	102.00 g	25.50 g	15.00 g	3.75 g	2.25 g	1.5 g
C	30%	89.25 g	38.25 g	15.00 g	3.75 g	2.25 g	1.5 g
D	40%	76.50 g	51.00 g	15.00 g	3.75 g	2.25 g	1.5 g
E	50%	63.75 g	63.75 g	15.00 g	3.75 g	2.25 g	1.5 g

**Table 2:** Size of white corundum with different meshes.

Longitudinal Size of Particle Size (μm)	200 mesh	320 mesh	600 mesh	1000 mesh
Maximum	126 ± 10 μm	82 ± 8 μm	39 ± 4 μm	20 ± 2 μm
Minimum	45 ± 5 μm	38 ± 4 μm	21 ± 2 μm	11 ± 1 μm
Average Value	81 ± 8 μm	52 ± 5 μm	28 ± 3 μm	15 ± 1 μm

## Data Availability

The original contributions presented in this study are included in the article. Further inquiries can be directed to the corresponding author.

## References

[1] Du Z., Yao D., Xia Y., Zuo K., Yin J., Liang H., Zeng Y.-P. (2020). Highly porous silica foams prepared via direct foaming with mixed surfactants and their sound absorption characteristics. Ceram. Int..

[2] Voigt C., Jäckel E., Taina F., Zienert T., Salomon A., Wolf G., Aneziris C.G., Le Brun P. (2017). Filtration Efficiency of Functionalized Ceramic Foam Filters for Aluminum Melt Filtration. Metall. Mater. Trans. B.

[3] Zhou W., Yan W., Li N., Li Y., Dai Y., Zhang Z., Ma S. (2019). Fabrication of mullite-corundum foamed ceramics for thermal insulation and effect of micro-pore-foaming agent on their properties. J. Alloys Compd..

[4] Hong Y., Wang Y., Li B., Pan G. (2019). Immobilizing nitrifying bacteria with Fe_2_O_3_-CaO-SiO_2_ porous glass-ceramics. Int. J. Appl. Glass Sci..

[5] Guo W., Hu T., Qin H., Gao P., Xiao H. (2021). Preparation and in situ reduction of Ni/SiC_x_O_y_ catalysts supported on porous SiC ceramic for ethanol steam reforming. Ceram. Int..

[6] Kim J., Ha J.-H., Lee J., Song I.-H. (2016). Optimization for Permeability and Electrical Resistance of Porous Alumina-Based Ceramics. J. Korean Ceram. Soc..

[7] Zhang F., Jia M., Wang J., Xu M., Wei C., Li W., Li Z. (2024). Freeze-Casting of Arbitrary-Shaped Porous Ceramics. Adv. Eng. Mater..

[8] Kotani M., Nishiyabu K., Matsuzaki S., Tanaka S. (2011). Processing of polymer-derived porous SiC body using allylhydridopolycarbosilane (AHPCS) and PMMA microbeads. J. Ceram. Soc. Japa..

[9] Hu L., Wang C.A., Huang Y., Sun C., Lu S., Hu Z. (2010). Control of pore channel size during freeze casting of porous YSZ ceramics with unidirectionally aligned channels using different freezing temperatures. J. Eur. Ceram. Soc..

[10] Krauss Juillerat F., Gonzenbach U.T., Elser P., Studart A.R., Gauckler L.J. (2011). Microstructural control of self-setting particle-stabilized ceramic foams. J. Am. Ceram. Soc..

[11] Wang Y., Wu T., Huang G. (2024). State-of-the-art research progress and challenge of the printing techniques, potential applications for advanced ceramic materials 3D printing. Mater. Today Commun..

[12] Man Y., Ding G., Xudong L., Xue K., Qu D., Xie Z. (2021). A review on porous ceramics with hierarchical pore structure by 3D printing-based combined route. J. Asian Ceram. Soc..

[13] Zhang F., Li Z., Xu M., Wang S., Li N., Yang J. (2022). A review of 3D printed porous ceramics. J. Eur. Ceram. Soc..

[14] Omatete O.O. (1991). Gelcasting-a new ceramic forming process. Cerem Bull.

[15] Young A.C., Omatete O.O., Janney M.A., Menchhofer P.A. (1991). Gelcasting of alumina. J. Am. Ceram. Soc..

[16] Xu J., Zhang Y., Gan K., Zhang X., Qu Y., Ma N., Yang J. (2015). A novel gelcasting of alumina suspension using curdlan gelation. Ceram. Int..

[17] Shahbazi H., Tataei M. (2019). A novel technique of gel-casting for producing dense ceramics of spinel (MgAl_2_O_4_). Ceram. Int..

[18] Mishra M., Bora J.J., Goswamee R.L. (2011). Improvement of the mechanical strength of alumina preforms by coating with montmorillonite/LDH gels. Appl. Clay Sci..

[19] Sun Z., Chen H., Meng X., Xiao G., Chen Z., Yi M., Zhang J., Liu W., Xu C. (2025). Influence of Embedding Microcapsules on Tribological Properties of Alumina Ceramics Prepared by Gel Casting. Materials.

[20] Caruso M.R., Calvino M.M., Šiler P., Cába L., Milioto S., Lisuzzo L., Lazzara G., Cavallaro G. (2025). Self-Standing Biohybrid Xerogels Incorporating Nanotubular Clays for Sustainable Removal of Pollutants. Small.

[21] Dong B., Yang M., Wang F., Hao L., Xu X., Wang G., Agathopoulos S. (2019). Porous Al_2_O_3_ plates prepared by combing foaming and gel-tape casting methods for efficient collection of oil from water. Chem. Eng. J..

[22] Zhou J., Wang C.A. (2013). Porous yttria-Stabilized Zirconia Ceramics Fabricated by Nonaqueous-Based Gelcasting Process with PMMA Microsphere as Pore-Forming Agent. J. Am. Ceram. Soc..

[23] Xia B., Wang Z., Gou L., Zhang M., Guo M. (2022). Porous mullite ceramics with enhanced compressive strength from fly ash-based ceramic microspheres: Facile synthesis, structure, and performance. Ceram. Int..

[24] Qi F., Xu X., Xu J., Wang Y., Yang J. (2014). A Novel Way to Prepare Hollow Sphere Ceramics. J. Am. Ceram. Soc..

[25] Yang Q., Zeng Z., Xu J., Zhang H., Ding J. (2006). Effect of La_2_O_3_ on Microstructure and Transmittance of Transparent Alumina Ceramics. J. Rare Earths.

[26] Park C.W., Yoon D.Y. (2000). Effects of SiO_2_, CaO_2_, and MgO Additions on the Grain Growth of Alu mina. J. Am. Ceram. Soc..

